# Karyological evidence of hybridogenesis in Greenlings (Teleostei: Hexagrammidae)

**DOI:** 10.1371/journal.pone.0180626

**Published:** 2017-07-05

**Authors:** Shota Suzuki, Katsutoshi Arai, Hiroyuki Munehara

**Affiliations:** 1 Graduate School of Environmental Science, Hokkaido University, Sapporo, Japan; 2 Usujiri Fisheries Station, Field Science Center for Northern Biosphere, Hokkaido University, Hakodate, Japan; 3 Graduate School of Fisheries Sciences, Hokkaido University, Hakodate, Japan; University of the Sunshine Coast, AUSTRALIA

## Abstract

Two types of natural hybrids were discovered in populations of three *Hexagrammos* species (Teleostei: Hexagrammidae) distributed off the southern coast of Hokkaido in the North Pacific Ocean. Both hybrids reproduce by hybridogenesis, in which the maternal haploid genome is transmitted to offspring without recombination and the paternal haploid genome is eliminated during gametogenesis. While natural hybrids are unisexual and reproduce hemiclonally by backcrossing with the paternal species (BC-P), artificial F_1_-hybrids between the pure species produce recombinant gametes. Thus, despite having the same genome composition, the natural hybrids and the F_1_-hybrids are not genetically identical. Here, to clarify the differences between both hybrids, we examined the karyotypes of the three *Hexagrammos* species, their natural hybrids, the artificial F_1_-hybrids, and several backcrosses. Artificial F_1_-hybrids have karyotypes and chromosome numbers that are intermediate between those of the parental species. Conversely, the natural hybrids differed from F_1_-hybrids by having several large metacentric chromosomes and microchromosomes. Since the entire maternal haploid genome is inherited by the natural hybrids, maternal backcrosses (BC-M) between natural hybrids and males of the maternal species (*H*. *octogrammus*; *Hoc*) have a hemiclonal *Hoc* genome with large chromosomes from the mother and a normal *Hoc* genome from the father. However, the large chromosomes disappear in offspring of BC-M, probably due to fissuring during gametogenesis. Similarly, microsatellite DNA analysis revealed that chromosomes of BC-M undergo recombination. These findings suggest that genetic factors associated with hemiclonal reproduction may be located on the large metacentric chromosomes of natural hybrids.

## Introduction

Hybridization between two closely related species can result in the production of fertile intermediate hybrids that produce gametes by meiosis and genetic recombination [1]. Conversely, hybridization between two distantly related species is typically associated with a disruption of normal meiosis due to pairing incompatibilities between homoeologous chromosomes. The consequences of hybridization primarily affect the genetic affinity between the two parental species, and most hybridization events result in the production of progeny with little, if any, survival potential, or even in sterility [2]. Gynogenesis, parthenogenesis and hybridogenesis are reproductive modes that have evolved in approximately 70 taxa in Animalia in order to avoid defective meiosis by incompatibilities between homoeologous chromosomes [3, 4, 5, 6]. In hybridogenesis, or hemiclonal reproduction, the haploid genome of one parent is transmitted to offspring without genetic recombination, while the other haploid genome is eliminated during gametogenesis [7, 8]. Unlike gynogenesis and parthenogenesis which involve clonal reproduction without the contribution of the spermatozoan genome, in hybridogenesis, the spermatozoan genome is used only in the replication of somatic cells and is eliminated from germ cells [9, 10]. Hybridogenesis has been reported in hybrids of freshwater fish, such as *Poeciliopsis* hybrids [11, 12], *Rutilus alburnoides* [13], and *Hypseleotris* [14]; the stick insect *Bacillus* [15]; the frog *Pelophylax esculentus* (*Rana esculentus*) [16]; and the marine fish *Hexagrammos* [17].

Based on karyological evidence, Cimino (1970) [18] was the first to propose that the paternal genome of *Poeciliopsis* hybrids is eliminated before the onset of meiosis and *Pelophylax esculentus* (*Rana esculentus*) [10, 19, 20]. A hypotheses for *Bacillus* hybrids indicates that the elimination of a genome takes place during polar bodies formation, i.e. at meiotic metaphases I and II [15]. Partial genome elimination at the onset of meiosis may avoid disrupting meiosis in homoeologous chromosomes. The progression of chromosomal pairing in germ cells is associated with checking each homologous chromosome during gametogenesis [21]. Differences between the karyotypes of two parental species typically results in the failure of chromosomal pairing due to elimination of the paternal genome at a genome-recognition checkpoint in germ cells. Consequently, (hemi-) clonal reproduction occurs very rarely, and only when there is sufficient genetic affinity between two parental species [22, 23]. This “balance hypothesis” means that the genetic divergence between the parental genomes has to be sufficiently large to affect meiosis in hybrids, but not so large that it causes a significant decrease in hybrid viability [24]. In the event that these conditions are met, (hemi-) clonal animals could be produced from artificial crosses between the parental species of the clone. For example, *Poeciliopsis* hybrids that originated from crosses between females of *P*. *monacha* and males of *P*. *lucida* were successfully propagated using hybridogenesis by backcrossing with males of *P*. *lucida* [12]. Hybrid embryos of *P*. *monacha* that were artificially fertilized by spermatozoa from *P*. *lucida* were aborted because the eggs did not retain sufficient yolk to develop until the larvae could feed independently [25]. This failure of larvae to develop was attributed to differences in yolk volume between the two parental species [25]. Stöck *et al*. (2010) [26] also failed to resynthesize the gynogenetic fish, *Poecilia formosa*, by hybridizing two parental species. The findings of these studies suggested the presence of specific differences in the genetic elements between the (hemi-) clonal genomes of natural hybrids and the genomes of the maternal species, but the genetic origin of unisexual reproduction remains unclear.

The genus *Hexagrammos* contains six species that are very common in the coastal waters of the North Pacific Ocean [27]. Of these species, the distribution of the one boreal species, the Masked greenling, *H*. *octogrammus* (hereafter *Hoc*), and two temperate species, the Fat greenling *H*. *otakii* (*Hot*) and Spottybelly greenling *H*. *agrammus* (*Hag*), overlap in the coastal areas of southern Hokkaido and Primorsky Krai of Russia [27, 28]. In these areas, two kinds of natural hybrids occur among the three *Hexagrammos* species [29, 30]. In both hybrids, *H*. *octogrammus* is the maternal progenitor. The two natural hybrids, which are all females produced from haploid eggs containing only the *Hoc* genome, reproduce by backcrossing with males of each paternal species, *Hag* or *Hot* [17]. The natural hybrids share closely related mtDNA haplotypes and possess a hemiclonal genome inherited from the maternal ancestor [31]. Interestingly, one hybrid (*Hoc/Hot* hybrid) was established by host switching with the other hybrid (*Hoc/Hag* hybrid), with *Hag* to *Hot* serving as the sperm-donor species [31]. Remarkably, while the artificial F_1_-hybrids produced by hybridizing two pure species have the same genome composition as the natural hybrids, the artificial F_1_-hybrids produced recombinant gametes [17], i.e., the reproductive system appeared to differentiate between the natural hybrids and artificial F_1_-hybrids in some way. These findings suggested that the *Hoc* genome of natural hybrids carried genetic factors that induced hybridogenesis, and that these factors were not present in the normal *Hoc* genome.

The genome of maternal backcrosses (BC-M), produced between a natural hybrid and a male of the maternal species (*Hoc*), comprises a diploid genome of *Hoc* genomes hemiclonally inherited from the hybrid and normally inherited from the male. Consequently, clarifying the reproductive mode employed by BC-M hybrids will provide important insights into the “balance hypothesis” or the aforementioned presence of genetic factors that induce hybridogenesis. If BC-M hybrids produce hemiclonal gametes, then the genetic factors that induce obligatory hybridogenesis must be carried by the Hoc* genome of natural hybrids (hereafter Hoc*, where * indicates the genetic factors inherited from the hybridogens). If BC-M hybrids produce recombinant gametes, then this suggests that genetic affinity is involved in the induction of hybridogenesis. Thus, in order to better clarify the genetic differences between natural hybrids and artificial hybrids, we compared the karyotypes of these species and several hybrids to determine the reproductive mode employed by BC-M hybrids and the mode of inheritance of the hemiclonal genome.

## Materials and methods

### Collection of parental fish

To obtain embryos for artificial fertilization, the three *Hexagrammos* species (*H*. *octogrammus; Hoc*, *H*. *agrammus; Hag*, and *H*. *otakii; Hot*) and the natural hybrids were caught using traps and/or fishing rods in the vicinity of Usujiri Fisheries Station (N41° 57’, E140° 58’) of the Field Science Center for Northern Biosphere, Hokkaido University in southern Hokkaido, Japan from 2011 to 2013. Species identification was performed based on the number of lateral lines, number of pairs of flaps on the head, and caudal fin shape according to Shinohara (1994) [32] and Nakabo (2013) [33]. Morphologically intermediate individuals of these three characters were separated as hybrids. The natural hybrids between *Hoc* and *Hag* (Hoc*/*Hag*) were identified by the number of lateral lines and flap pairs: lack of the forth lateral line and one flap on the posterior region of the head. The natural hybrids between *Hoc* and *Hot* (Hoc*/Hot) were identified by the number of flap pairs and the shape of the caudal fin: lack of a flap pair on the posterior region of the head and caudal fins with a rounded margin. Hybrid identification based on these morphological characteristics was genetically verified by Kimura-Kawaguchi *et al*. (2014) [17].

Before artificial fertilization, the captured live fishes were kept in 500 L tanks containing concrete blocks that served as hiding places. Fresh seawater was pumped into the tank to ensure that the water temperature was the same as that in the natural environment. Fishes were fed daily with Japanese anchovy, krill and artificial pellets (Otohime, Nishinmarubeni Co., Japan).

**Fig 1:**
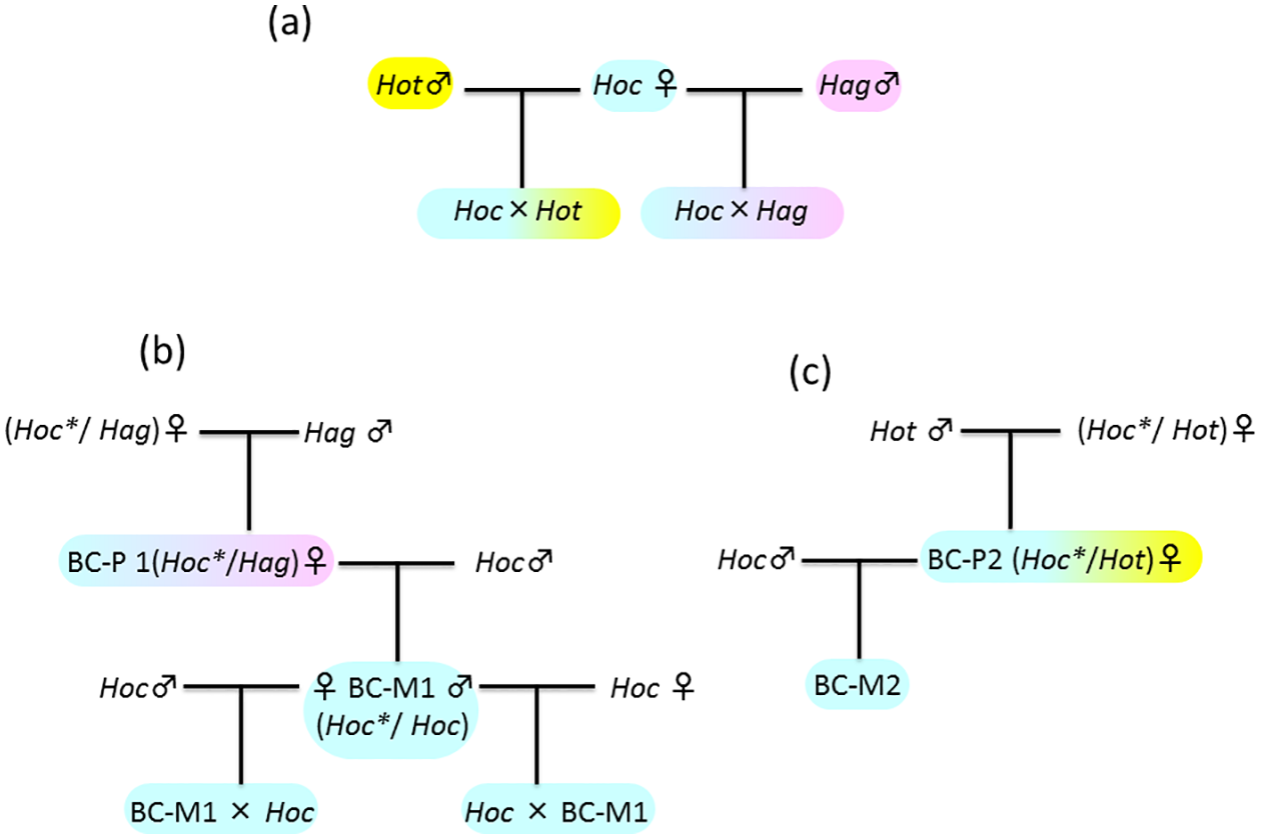
The lineages of specimens used in this study. (a) artifial F_1_-hybrids, (b) paternal backcross lineage, (c) maternal backcross lineage. All abbreviated species signs were referred to text. Yellow, light blue and purple of species signs indicate the *H*.*otakii*, *H*. *octogrammus* and *H*. *aggrammus* genome, respectively. Uncolored signs indicate species that not observed in this study.

Chromosome spreads were prepared from embryos of *Hoc*, *Hag*, *Hot*, the two natural hybrids (*Hoc/Hag and *Hoc/Hot), artificial F_1_-hybrids (*Hoc* × *Hag* and *Hoc* × *Hot*), and several backcrosses, as described below. The generalized format used to represent the hybrid crosses in this study was as follows: A × B and A/B, where A is the female species, B is the male species, and “/” and “×” indicate natural hybrids and artificial crossing, respectively. F_1_-hybridization between species is depicted here as *Hoc* × *Hag* and *Hoc* × *Hot*. Different symbols are used to represent these hybrids, because the natural hybrids reproduce hemiclonally by backcrossing with paternal species, whereas F_1_-hybrids resulting from hybridization between species produced recombinant gametes [17]. In such a situation, paternal backcrosses of natural hybrids are described as BC-P1: (Hoc*/Hag) × *Hag* and BC-P2: (Hoc*/Hot) × *Hot*, and they thoroughly correspond with the natural hybrids Hoc*/Hag and Hoc*/Hot, respectively. In the same way, maternal backcrosses of natural hybrids are referred to as BC-M1: (Hoc*/Hag) × *Hoc* and BC-M2: (Hoc*/Hot) × *Hoc*. Both the artificial hybrids comprise a Hoc* genome and a *Hoc* genome. When they matured at 2 years, it was found that both sexes of BC-M hybrids were produced, which meant that both BC-M1 × *Hoc* and *Hoc* × BC-M1 could be crossed in order to observe the resulting karyotypes and to investigate their respective reproductive modes. The lineages of specimens used for observation of a chromosome were indicated in Fig 1.

### Artificial fertilization

Ovulation was confirmed by applying gentle pressure to the abdomen. When the females ovulated, the eggs were evenly divided into separate petri dishes. Milt was collected with a hematocrit tube. The amount and time of handling of the fish was kept to a minimum to ameliorate suffering and distress, and collecting eggs and spermatozoa were conducted with anesthesia (MS-222). Sperm motility was checked by adding seawater under a microscope (× 40). After checking, sperm with high motile activity were used for artificial fertilization. The eggs were fertilized with a sufficient quantity of semen (5–10 μl) and fertilization was conducted using a standard dry method. After thinly spreading the eggs, spermatozoa were added with gentle mixing using a spatula. The fertilized eggs were then incubated at room temperature for several minutes to complete fertilization, followed by incubation at 11–15°C for 3 to 7 days until the eyes appeared. For identification purposes, all of the parental fish used for artificial fertilization were identified using internal electric tags or external plastic tags.

### Collection of egg masses from natural spawning grounds

Most of the embryos were prepared by artificial fertilization, but some were collected from natural breeding sites. Eggs of Hoc*/Hot natural hybrids were collected from *Hot* spawning grounds on the western side of Usujiri fishing port by SCUBA from October to December, 2011 to 2013. The eggs of natural hybrids deposited in *Hot* territories were identified based on the color of the oil droplets in the yolk using the method of Kimura *et al*. (2007) [34]. *Hag* egg masses were collected in the coastal waters of Sado Island (N38° 04’, E138° 14’) using SCUBA in December 2011. These egg masses were also incubated at 15°C until the eyed embryo stage.

### Ethics statement

All specimens were collected in accordance with the national legislation of the countries concerned.

The experimental procedures involving fish were approved by the Institutional Animal Care and Use Committee (The Regulations of Animal Experimentation at Hokkaido University), Permit number 26–1, according with directives from the Ministry of Education, Culture, Sports, Science and Technology, Japan.

### Chromosome preparations and analyses

Karyotypes of *Hot* and *Hag* have been studied by Matsumiya *et al*. (1980) [35] and Nishikawa and Sakamoto (1982) [36], and the modal chromosome number of *Hoc* has been estimated by Makino (1937) [37]. In the present study, including the pure species, a total of 43 egg batches were prepared for chromosome analysis. The number of egg batches from each species (strains) were 5 *Hot*, 5 *Hag*, 6 *Hoc*, 3 *Hoc* × *Hag*, 3 *Hoc* × *Hot*, 5 Hoc*/Hag, 5 *Hoc* × *Hot*, 3 BC-M1, 3 BC-M2, 2 BC-M1 × *Hoc* and 3 *Hoc* × BC-M1. Yolk was aseptically removed from eyed embryos in 0.9% NaCl solution with tweezers under a microscope (SZX, Olympus Co., Japan). The resulting embryos were then kept in a 0.9% NaCl solution containing 0.0025% colchicine for 1.5 to 2.5 h, before being placed into a 0.075 M KCl hypotonic solution for 30–60 min at room temperature and then fixed with a Carnoy solution (methanol:acetic acid = 3:1) in microtubes overnight. After resuspending the cells in an aqueous solution using a micropipette, chromosome spreads were obtained by dripping the aqueous solution on a glass microscope slide from a height of 30 cm. The chromosome spreads were then stained with 4% Giemsa in phosphate buffer (pH = 6.8) for 30 min.

Chromosomes were observed under a microscope (BX51, Olympus Co.). The karyotypes were determined by chromosomes that were reconstructed from Giemsa-stained metaphase plates using a microscope equipped with a digital camera (VB-7010, Keyence Co., Japan). According to Levan *et al*. (1964) [38], chromosomes were classified as *m* = metacentric; *sm* = submetacentric; *st* = subtelocentric and *t* = telocentric. The fundamental number (NF) was calculated by assigning 2 arms for *m* and *sm* and 1 arm for *st* and *t*. A pair of homologous chromosomes was determined by the relative length of the short and long arms of each chromosome.

### Reproductive mode of BC-M by microsatellite DNA analyses

In order to investigate the reproductive mode of BC-M, 2 BC-M1 × *Hoc* and 3 *Hoc* × BC-M1, batches were genotyped by microsatellite DNA analysis. The eggs were maintained in incubation tanks with running filtered seawater at a temperature of 13–16°C. Ten larvae were removed from each batch just after hatching, and preserved in 99% ethanol at -10°C until genetic analysis.

For genotyping offspring from batches of BC-M1 × *Hoc* and *Hoc* × BC-M1, three highly polymorphic microsatellite DNA loci (*Hexoc 6*, *14* and *20*) were selected, as these have been used in previous studies [17, 31]. Total genomic DNA was extracted using a Quick Gene DNA tissue kit S (Fujifilm, Japan) according to the manufacturer’s instructions and stored in a refrigerator at 4°C until use. PCR mixes contained 6.25 μL of EmeraldAmp^®^ PCR Master Mix (Takara Bio Inc., Otsu, Japan), 6.25 pmol of each primer, 0.5 μL of template DNA (50–100 ng/μL), and water to a final volume of 12.75 μL. PCR reactions were performed in a PCR Thermal Cycler (Takara Bio Inc.) with profiles consisting of an initial denaturation step of 120 s at 94°C, followed by 25–30 cycles of 30 s at 94°C, 30 s at the annealing temperature, and 120 s at 72°C. The forward and reverse primer sequences and the annealing temperatures for *Hexoc 6*, *14* and *20* were forward-GGATAGTTTGTTCCTGTCAG, reverse-AAATGTTTGTCCCCAAACCC and 50°C, forward-CGGGGTAGTGAAGCATGAT, reverse-TTTTGTACTTGTGTTTTCCT and 54°C, and forward-GAGGGACAGCAGGCAGAGAA, reverse-ATGCAGCTACACATAGAGTGT and 60°C, respectively. The genotypes were verified by separating the PCR products on 6–10% polyacrylamide gels stained with SYBR^®^ Green II (Takara Bio Inc.).

## Results

### Karyotypes of three *Hexagrammos* species

**Fig 2:**
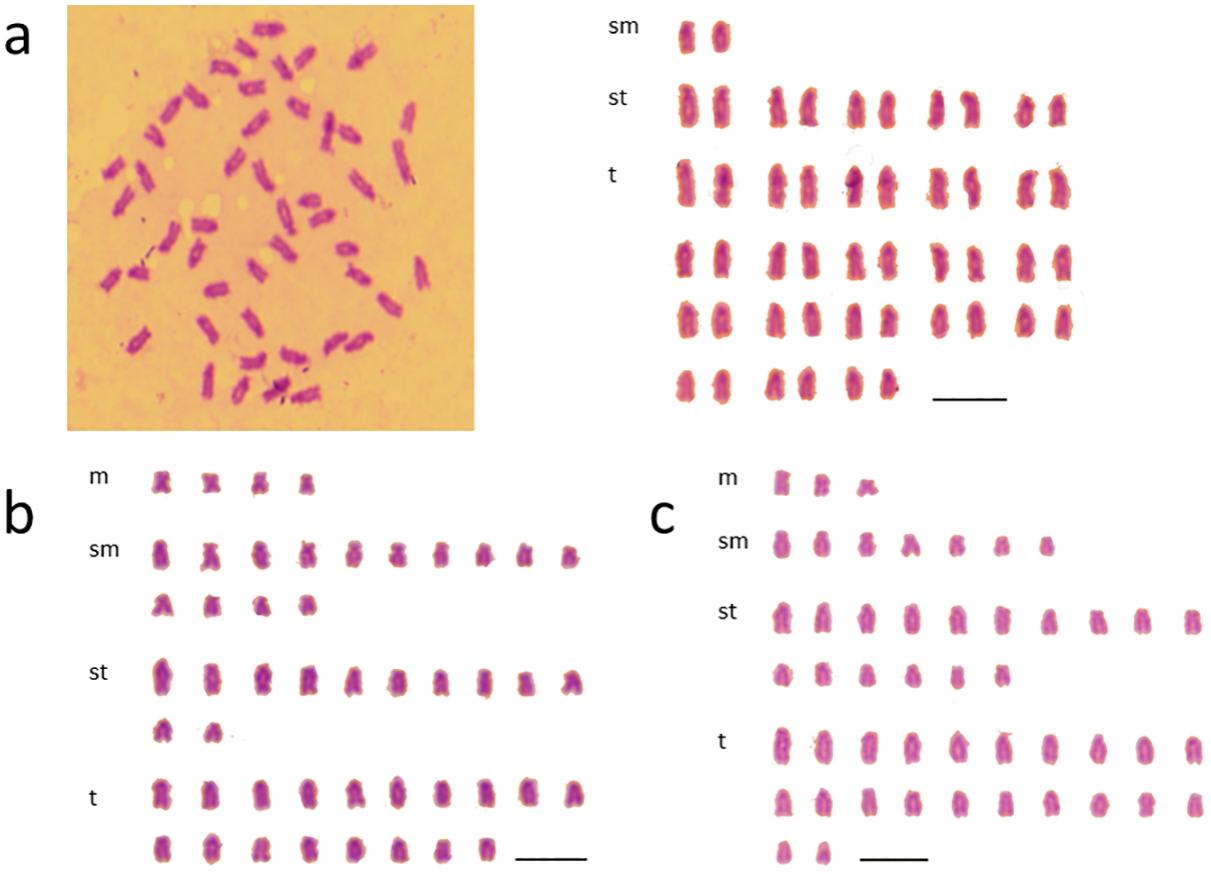
Mitotic metaphase chromosome spread and karyotypes. (a) *H*. *octogrammus*, (b) F_1_-hybrid (*Hoc* × *Hag*), (c) F_1_-hybrid (*Hoc* × *Hot*). Spreads of F_1_-hybrids were showed in S1.

Among 62 metaphases from six batches of *H*. *octogrammus* (*Hoc*), the modal chromosome number was 2n = 48 (Table 1). The karyotype was determined as 2*sm* + 10*st* + 36*t*, and the NF was 50 arms (Fig 2a).

**Table 1:** Karyological observations of the three *Hexagrammos* species, and the natural hybrids and the artificial hybrids. Yellow cells indicate the most frequent chromosome numbers of each species and hybrid.

Species	Number of batches observed	Number of cells observed	Number of individuals	Number of chromosomes observed in a preparation (excluding micro chromosomes)							Type	Karyotype				Number of centric fusion	Number of micro chromosomes	NF	Number of chromosomes (2n)
				∼43	44	45	46	47	48	49		*m*	*sm*	*st*	*t*				
*H*. *octogrammus*	6	62	10		2	2	4	8	45	1		0	2	10	36			50	48
*H*. *agrammus*	5	9	5				2	1	6			8	26	14	0			82	48
*H*. *otakii*	5	22	30				4	4	14			6	12	22	8			66	48
*Hoc×Hag*	3	120	13	2	1	3	2	10	99	3		4	14	12	18			66	48
*Hoc×Hot*	3	121	12	3	1	6	7	12	86	6		3	7	16	22			58	48
*Hoc/Hag*	5	140	7		8	13	53	7	7		Type 1	6	14	8	18	2		66	46
			5	9	3	27	2				Type 2	7	14	8	16	3		66	45
			1	1				10			Type 3	7	14	8	16	3	2	66	47
*Hoc/Hot*	5	151	10		7	13	30	8	1		Type 1	5	7	12	22	2		58	46
			6	20	9	45	2			2	Type 2	6	7	12	20	3		58	45
			2			2	8	4			Type 3	6	7	12	20	3	1	58	46
BC-M1: (*Hoc*/*Hag*)×*Hoc*	3	90	7	2	2	4	42				Type 1	2	2	6	36	2		50	46
			3	6	1	25					Type 2	3	2	6	34	3		50	45
			1		1	3	1	6			Type 3	3	2	6	34	3	2	50	47
BC-M2: (*Hoc*/*Hot*)×*Hoc*	3	101	7	1	6	41	1	1			Type 1	3	2	6	34	3		50	45
			3	3	44	4					Type 2	4	2	6	32	4	1	50	45
BC-M1×*Hoc*	2	80	8		1			8	69	2		0	2	8	38			50	48
*Hoc*×BC-M1	3	59	6			1	7	5	45	1		0	2	8	38			50	48

Among nine metaphases from five batches of *H*. *agrammus* (*Hag*), the modal chromosome number was 2n = 48. The karyotype was 8*m* + 26*sm* + 14*st*, and NF was 82 arms.

Among 22 metaphases from five batches of *H*. *otakii* (*Hot*), the modal chromosome number was 2n = 48. The karyotype was 6*m* + 12*sm* + 22*st* + 8*t*, and the NF was 66 arms.

### Karyotypes of artificial F_1_-hybrids

Among 120 metaphases from three batches of *H*. *octogrammus* × *H*. *agrammus* (*Hoc* × *Hag*), the modal chromosome number was 2n = 48. The karyotype was 4*m* + 14*sm* + 12*st* + 18*t*, and the NF was 66 arms (Fig 2b, S1a Fig).

Among 121 metaphases from three batches of *H*. *octogrammus* × *H*. *otakii* (*Hoc* × *Hot*), the modal chromosome number was 2n = 48. The karyotype was 3*m* + 7*sm* + 16*st* + 22*t*, and the NF was 58 arms (Fig 2c, S1b Fig). These results showed that the karyotypes of both F_1_-hybrids were intermediate between each of the two parental species.

### Karyotypes of the natural hybrids

Among 140 metaphases from five batches of *H*. *octogrammus* /*H*. *agrammus* (Hoc*/Hag), two or three large metacentric chromosomes were observed in all of the cells (Table 1). These large chromosomes were inferred to comprise two chromosomes that had become joined, and which are mentioned in the discussion. In addition, two microchromosomes were observed in some cells. The modal chromosome numbers in these hybrids were 2n = 45–47 and the karyotypes were divided into three types as follows:

(Here and below, the numerals in parenthesis represent the number of large-size metacentric chromosomes).

**Fig 3:**
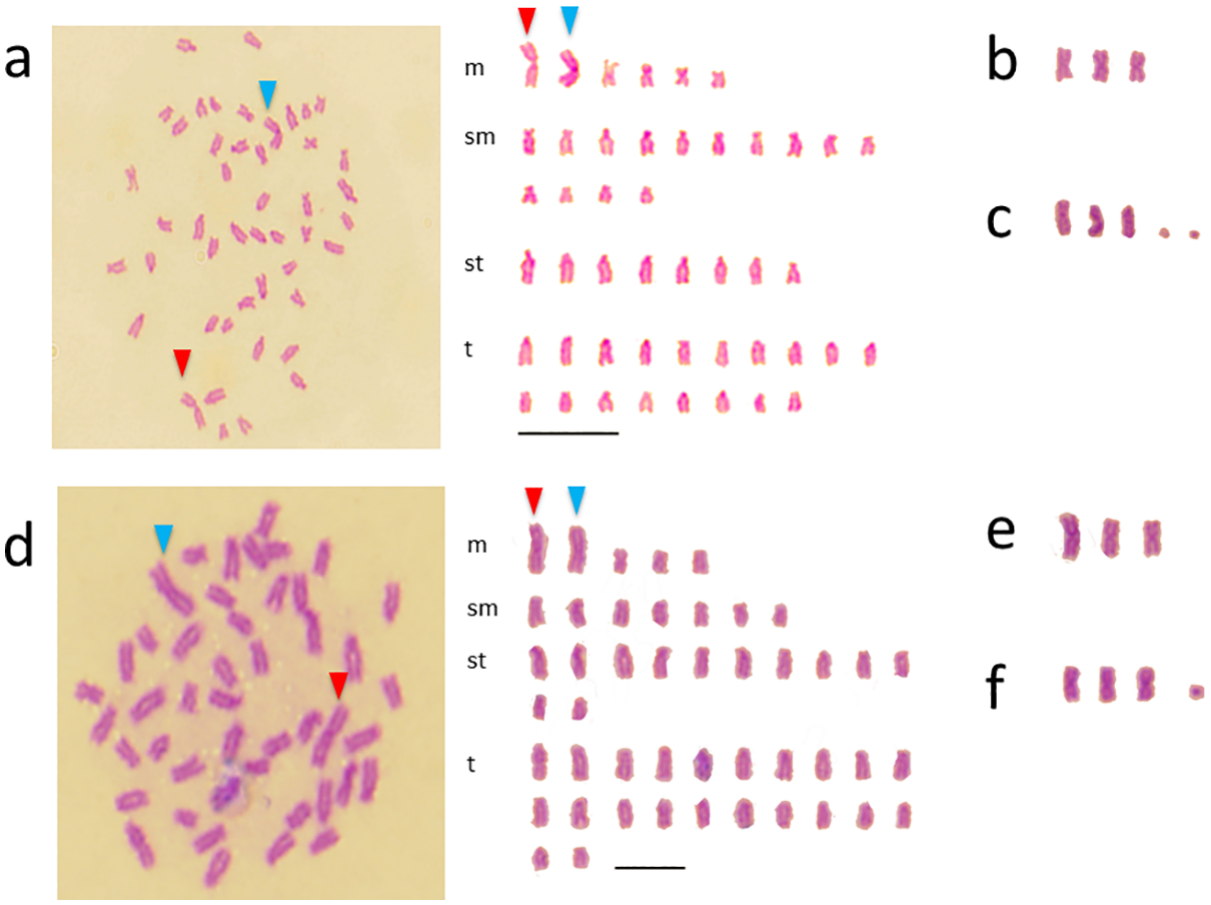
Mitotic metaphase chromosome spreads and karyotypes of natural hybrids. (a) Hoc*/*Hag* type 1, (b) large metacentric chromosomes of Hoc*/*Hag* type 2, (c) large metacentric chromosomes and microchromosome of Hoc*/*Hag* type 3; (d) Hoc*/*Hot* type 1, (e) large metacentric chromosomes of Hoc*/*Hot* type 2, (f) large metacentric chromosomes and microchromosome of Hoc*/*Hot* type 3. Arrow heads showed large metacentric chromosomes. The remaining spreads of Hoc*/*Hag* and Hoc*/*Hot* were showed in S2 and S3, respectively.

Type 1: 6(2)*m* + 14*sm* + 8*st* + 18*t*, 46 chromosomes and 66 arms (Fig 3a).
Type 2: 7(3)*m* + 14*sm* + 8*st* + 16*t*, 45 chromosomes and 66 arms (Fig 3b, S2a Fig).
Type 3: 7(3)*m* + 14*sm* + 8*st* + 16*t*, 47 chromosomes including 2 microchromosomes and 66 arms (Fig 3c, S2b Fig).

In Hoc*/Hot crosses, among 151 metaphases from five batches, two or three large metacentric chromosomes and one microchromosome were observed in all of the cells. The modal chromosome numbers were 2n = 45 or 46, and the karyotypes were divided into three types.

Type 1: 5(2)*m* +7 *sm* + 12*st* + 22*t*, 46 chromosomes and 58 arms (Fig 3d).
Type 2: 6(3)*m* + 7*sm* + 12*st* + 20*t*, 45 chromosomes and 58 arms (Fig 3e, S3a Fig).
Type 3: 6(3)*m* + 7*sm* + 12*st* + 20*t*, 46 chromosomes including one microchromosome and 58 arms (Fig 3f, S3b Fig).

### Karyotypes of maternal backcross; Natural hybrid × *H*. *octogrammus* (BC-M)

Among 90 metaphases from three batches of BC-M1 (Hoc*/*Hag*×*Hoc*) also, two or three large metacentric chromosomes and two microchromosomes were observed. The modal chromosome number was 2n = 45–47, and the karyotypes were divided into three types.

**Fig 4:**
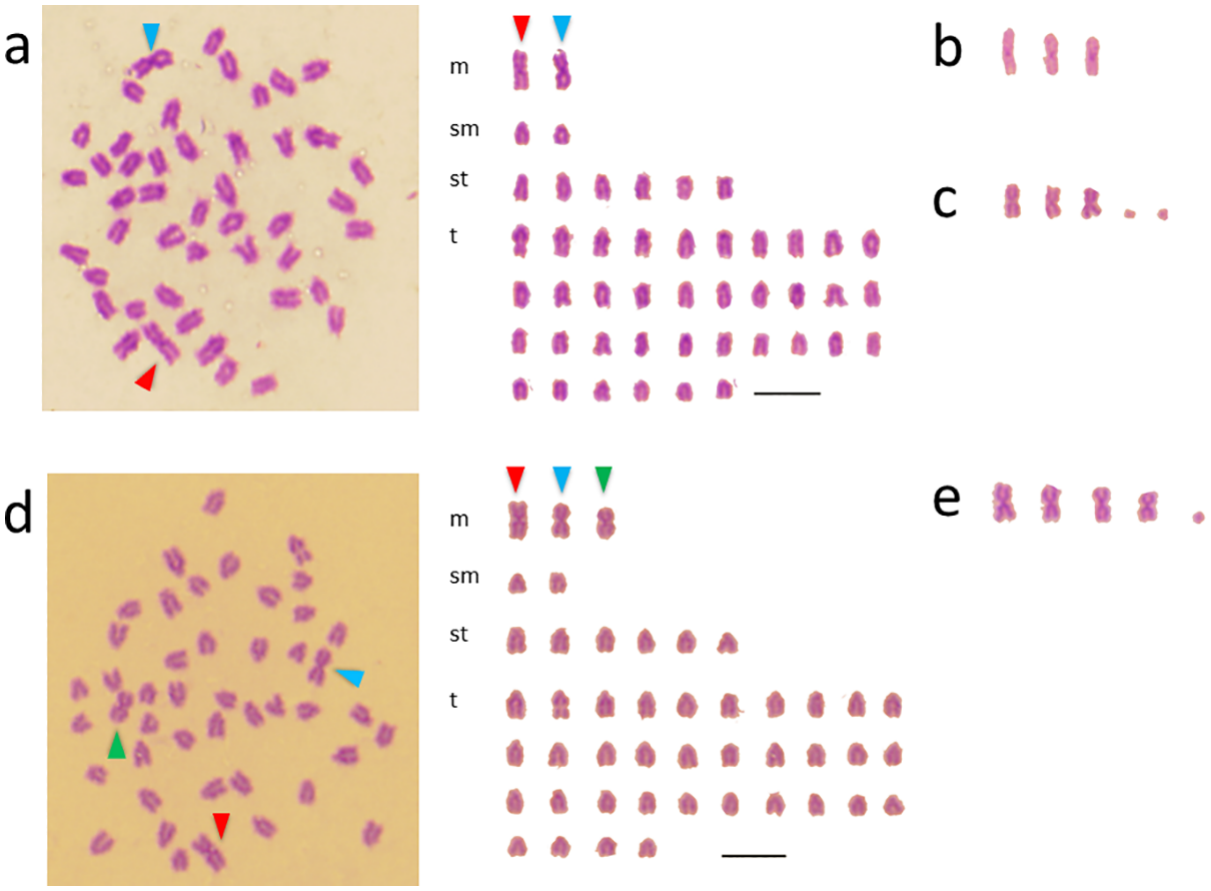
Mitotic metaphase chromosome spreads and karyotypes of maternal backcross. (a) BC-M1 ((Hoc*/*Hag*) × *Hoc*) type 1, (b) large metacentric chromosomes of BC-M1 ((Hoc*/*Hag*) × *Hoc*) type 2, (c)) large metacentric chromosomes and microchromosome of BC-M1 ((Hoc*/*Hag*) × *Hoc*) type 3; (d) BC-M2 ((Hoc*/*Hot*) × *Hoc*) type 1, (e)) large metacentric chromosomes and microchromosome of BC-M2 ((Hoc*/*Hot*) × *Hoc*) type 2. Arrow heads showed large metacentric chromosomes. The remaining spreads of BC-M1 ((Hoc*/*Hag*) × *Hoc*) and BC-M2 ((Hoc*/*Hot*) × *Hoc*) were showed in S4 and S5, respectively.

Type 1: 2(2)*m* + 2*sm* + 6*st* + 36*t*, 46 chromosomes and 50 arms (Fig 4a).
Type 2: 3(3)*m* + 2*sm* + 6*st* + 34*t*, 45 chromosomes and 50 arms (Fig 4b, S4a Fig).
Type 3: 3(3)*m* + 2*sm* + 6*st* + 34*t*, 47 chromosomes including two microchromosomes and 50 arms (Fig 4c, S4b Fig).

Among 101 metaphases from three batches of BC-M2 (Hoc*/*Hot* × *Hoc*), three or four large metacentric chromosomes and one microchromosome were observed in all the cells. The modal chromosome number was 2n = 45, and the karyotypes were divided into 2 types.

Type 1: 3(3)*m* + 2*sm* + 6*st* + 34*t*, 45 chromosomes and 55 arms (Fig 4d).
Type 2: 4(4)*m* + 2*sm* + 6*st* +32*t*, 45 chromosomes including one microchromosome and 50 arms (Fig 4e, S5a Fig).

These results showed that, of the *Hoc* genomes, although the genome compositions of both BC-M1 and BC-M2 are diploid, the karyotypes differed slightly from normal *Hoc*.

### Karyotype of BC-M1 × *Hoc*

**Fig 5:**
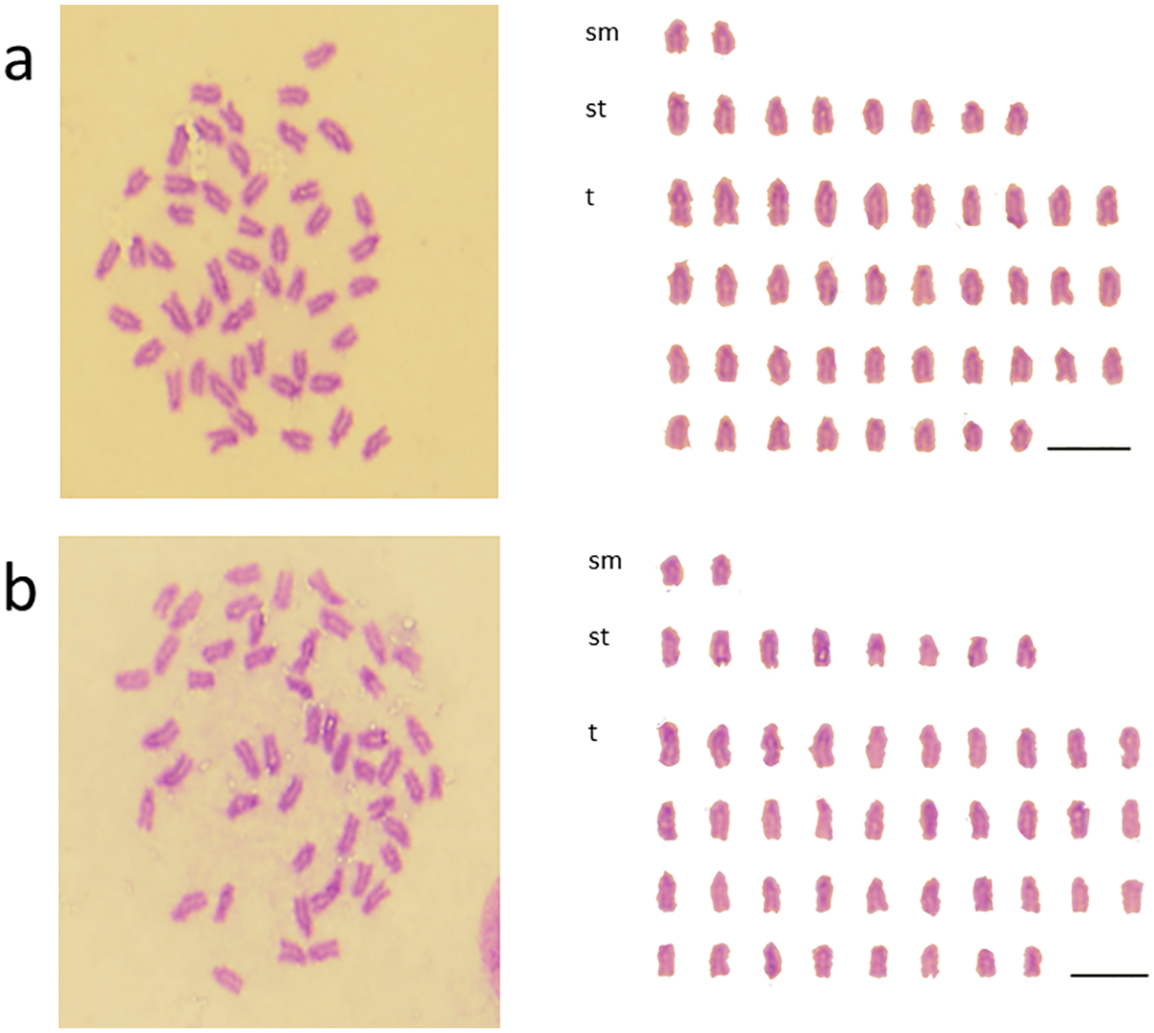
Mitotic metaphase chromosome spreads and karyotypes. (a) BC-M1 × *Hoc*; (b) *Hoc* × BC-M1.

Among 80 metaphases from two batches of BC-M1 × *Hoc*, none of the large metacentric chromosomes or microchromosomes were observed in any of the cells. The modal chromosome number was 2n = 48, the karyotype was 2*sm* + 8*st* + 38*t*, and the NF was 50 arms (Fig 5a).

### Karyotype of *Hoc* × BC-M1

Among 59 metaphases from three batches of *Hoc* × BC-M1, none of the large metacentric chromosomes or microchromosomes were observed in any of the cells. The modal chromosome number was 2n = 48, the karyotype was 2*sm* + 8*st* + 38*t*, and the NF was 50 arms (Fig 5b). BC-M1 × *Hoc* and *Hoc* × BC-M1 exhibited the same karyological morphology. The genomic composition of these hybrids was a diploid *Hoc* genome and a Hoc* genome, which was the same as BC-M; however, the karyotype of BC-M1 × *Hoc* and *Hoc* × BC-M1 differed from both BC-M and the pure *Hoc* species.

### Reproductive mode of BC-M

Ten larvae from each batch were genotyped for three microsatellite DNA loci; however, since the BC-M specimens have homogeneous alleles, only two microsatellite DNA loci were assayed in this group (Table 2). When both of the maternal alleles were found in a batch, the hybrid was regarded as being capable of producing recombinant gametes. When all of the offspring in a batch shared one of the two maternal alleles, BC-M were considered to produce hemiclonal haploid gametes (hybridogenesis).

**Table 2:** Reproductive mode of maternal backcross (BC-M) inferred from genotyping offspring of (*Hoc* x BC-M; Cross 1–3) and (BC-M x *Hoc*; Cross 4 and 5) using microsatellite DNA. In BC-M retaining two *Hoc* genome sets, alleles (orange) from hemiclonal hybrids (grandmother: *Hoc/Hag*) were inherited by offspring after recombination. Alleles (blue) from fathers (*Hoc*) are colored to facilitate discrimination.

*Hoc* x BC-M													
Cross 1	Loci	Genotypes of		Genotypes of Offspring									
		Mother	Father	1	2	3	4	5	6	7	8	9	10
Mother ID; C197	*Hexoc 6*	112/114	106/134	106/112	112/134	106/112	114/134	112/134	106/114	106/112	114/134	114/134	106/112
Father ID; 121020	*Hexoc 14*	116/128	128/128	BC-M; homologous allele									
	*Hexoc 20*	112/112	108/118	108/112	108/112	108/112	108/112	112/118	108/112	112/118	108/112	108/112	112/118
Cross 2	Loci	Genotypes of		Genotypes of Offspring									
		Mother	Father	1	2	3	4	5	6	7	8	9	10
Mother ID; 898206	*Hexoc 6*	102/118	106/132	106/118	102/132	118/132	106/118	102/106	118/132	118/132	102/132	106/118	102/132
Father ID; 15224	*Hexoc 14*	128/132	128/128	BC-M; homologous allele									
	*Hexoc 20*	110/116	108/114	114/116	108/110	108/110	108/110	114/116	108/116	110/114	110/114	108/116	114/116
Cross 3	Loci	Genotypes of		Genotypes of Offspring									
		Mother	Father	1	2	3	4	5	6	7	8	9	10
Mother ID; 898206	*Hexoc 6*	102/118	106/112	106/118	102/106	102/112	102/112	112/118	106/118	106/118	112/118	102/106	106/118
Father ID; 149729	*Hexoc 14*	128/132	128/128	BC-M; homologous allele									
	*Hexoc 20*	110/116	108/114	110/114	114/116	108/116	114/116	108/110	110/114	108/110	108/116	110/114	110/114
BC-M x *Hoc*													
Cross 4	Loci	Genotypes of		Genotypes of Offspring									
		Mother	Father	1	2	3	4	5	6	7	8	9	10
Mother ID; 603907	*Hexoc 6*	116/120	100/120	116/120	120/120	120/120	110/116	100/120	100/116	116/120	100/120	100/120	100/120
Father ID; 135641	*Hexoc 14*	122/124	116/124	124/124	122/124	122/124	124/124	116/124	124/124	122/124	116/124	122/124	122/124
	*Hexoc 20*	108/116	110/116	108/110	108/110	108/116	108/116	108/110	110/116	110/116	110/116	108/116	108/116
Cross 5	Loci	Genotypes of		Genotypes of Offspring									
		Mother	Father	1	2	3	4	5	6	7	8	9	10
Mother ID; 136762	*Hexoc 6*	106/110	104/110	104/106	104/106	110/110	104/106	106/110	104/106	104/106	110/110	106/110	106/110
Father ID; 128801	*Hexoc 14*	116/128	112/130	116/130	112/128	112/128	112/128	112/128	112/128	116/130	116/130	116/130	128/130
	*Hexoc 20*	108/112	112/114	112/112	112/114	112/112	108/112	108/112	112/114	108/112	108/112	112/114	108/112

In all three batches of *Hoc* × BC-M1 and the two batches of *Hoc* × BC-M1, both of the alleles from BC-M were observed (Table 2), suggesting that both males and females of BC-M1 were capable of producing recombinant gametes. BC-M comprise the Hoc* genome and the *Hoc* genome, which they inherited from a natural hybrid and a normal *Hoc*, respectively. This result suggested that hybridogenesis was not induced when individuals were diploid for conspecific genomes.

## Discussion

### Karyotypes of three *Hexagrammos* species and artificially produced F_1_-hybrids

The results obtained for the pure species in the present study corroborated those of previous studies [35, 36, 37]. The karyotypes of the three *Hexagrammos* species were observed to have diversified, especially that of *Hoc*, which was comparatively larger than the other two species. For example, while *Hot* and *Hag* have 6 and 8 metacentric chromosomes that are not found in *Hoc*, the latter has 36 telocentric chromosomes while *Hot* has only 8 and *Hag* has none. *Hag* and *Hot* have also been shown to be genetically closer to each other than either is to *Hoc* [32, 39], and flow cytometry analysis has shown that *Hoc* has 1.5-times as much genetic material as *Hag* and *Hot* [17]. The more marked karyological differences between *Hoc* and the other two species observed in this study therefore correspond to these previous observations of genetic differences among these three species.

### Robertsonian translocation in the natural hybrids

Natural hybrids (Hoc*/*Hot* and Hoc*/*Hag*) hemiclonally produce haploid eggs with a maternally derived genome, whereas artificial F_1_-hybrids (*Hoc* × *Hag* and *Hoc* × *Hot*) produce recombinant gametes by meiosis [17]. Although the karyotypes of both F_1_-hybrids were intermediate between the two parental species, the karyotypes of both natural hybrids differed from those of each F_1_-hybrid and comprised large metacentric chromosomes. BC-M, which have Hoc* and *Hoc* genomes, exhibited karyotypes that comprised two to three large metacentric chromosomes. The large metacentric chromosomes appear to have been transmitted from the Hoc* genome of the natural hybrid. Since karyotypes of *Hoc* and *Hag* were 0*m* + 2*sm* + 10*st* + 36*t* and 8*m* + 26*sm* + 14*st* + 0*t*, respectively, the numerical mean was 4*m* + 14*sm* + 12*st* + 18*t*, which corresponded to the karyotype of the F_1_-hybrid, *Hoc* × *Hag*. If the two united chromosomes were formerly subtelocentric, then 2*st* would be counted as 1*m*. Type 1 of the hemiclonal hybrid Hoc*/*Hag*, which has two large metacentric chromosomes, was 6(2)*m* +14*sm* + 8*st* + 18*t* and was applied to this conversion. In the case of Type 2, if one of the three united chromosomes was a previously submetacentric or telocentric chromosome, then 2*t* would be counted as 1*m*. Type 2 of Hoc*/*Hag* was 7(3)*m* +14*sm* + 8*st* + 16*t*, which was applied to this conversion, as were the karyotypes of another hemiclonal hybrid (Hoc*/*Hot*).

The large metacentric chromosomes were concluded to have united based on the finding that the two chromosomes fused near the centromere. This type of large metacentric chromosome is considered to correspond to “centric-fusion” and this is referred to as a Robertsonian translocation [40](Lajus 2007). Robertsonian translocation in fish was first described in the Reticulated dascyllus, *Dascyllus reticulates* [41]. In *Hexagrammos*, the differences in the karyotypes of natural hybrids and artificial F_1_-hybrids are considered to reflect the different reproductive modes.

### Reproductive mode for BC-M and fission of Robertsonian translocation

The Hoc* genome is identical to the haploid karyotype of the *Hag* genome subtracted from the Hoc*/Hag karyotype of natural hybrids obtained through hemiclonal reproduction. Type 1 and 2 karyotypes of Hoc*/Hag are calculated as 2(2)*m* + 1*sm* + 1*st* + 18*t* and 3(3)*m* + 1*sm* + 1*st* + 16*t*, respectively. The karyotypes of BC-M2, which were produced by adding hemiclonal eggs and haploid sperm from *Hoc*, can be represented as 2(2)*m* + 2*sm* + 6*st* + 36*t* and 3(3)*m* + 2*sm* + 6*st* + 34*t*. The former is identical to the Type 1 karyotype of BC-M2, while the latter is somewhat similar to the Type 3 karyotype of BC-M2. Specifically, the slight difference between the latter and the Type 3 karyotype of BC-M2 is the presence of a large metacentric chromosome consisting of 2*st*. Interestingly, while the large metacentric chromosomes were inherited by the somatic cells of BC-M, they disappeared in BC-M1 × *Hoc* and *Hoc* × BC-M1 crosses. These findings imply that the large chromosomes had been fissured, probably in germ line cells.

**Fig 6:**
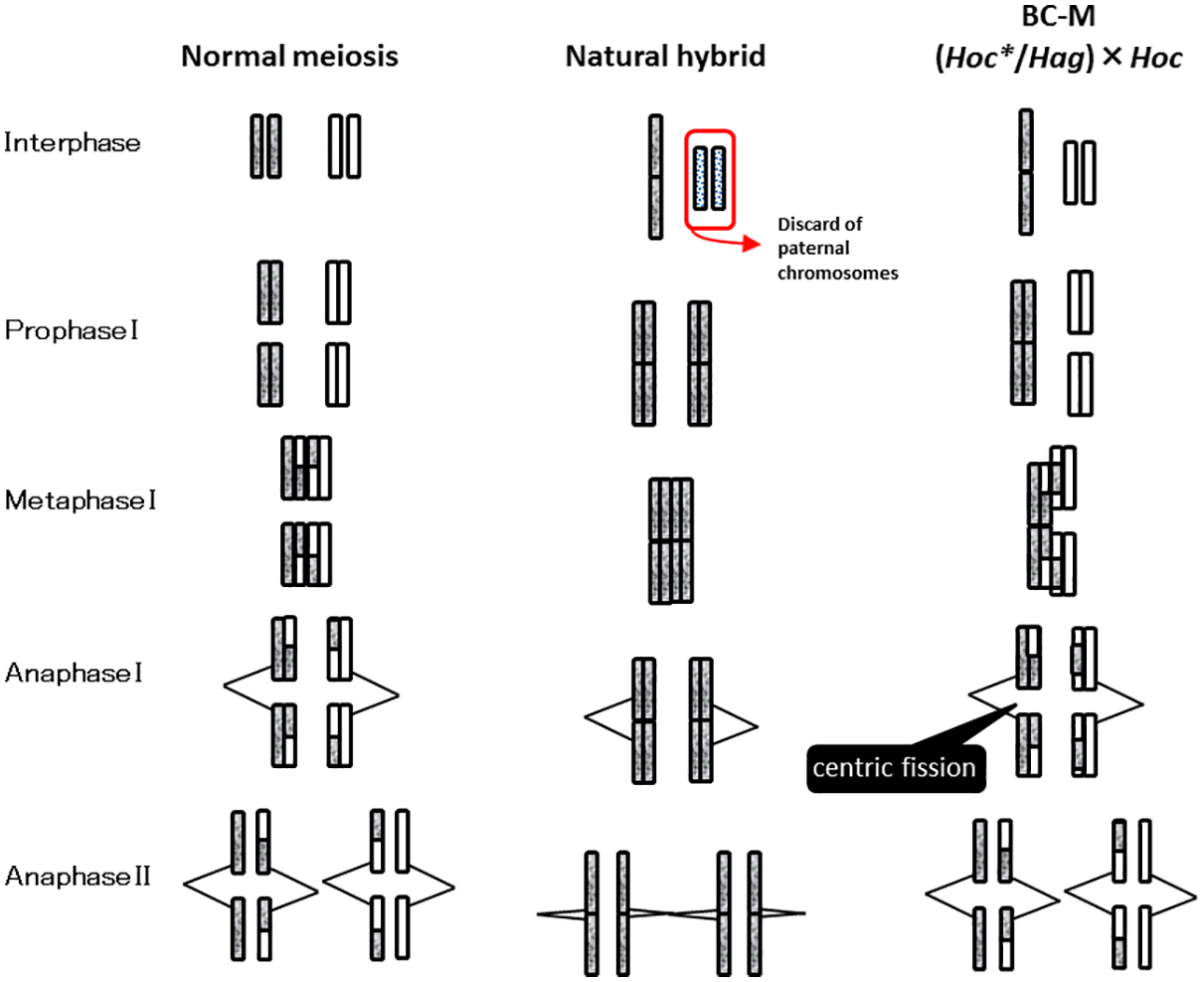
Schema of meiosis of natural hybrids and BC-M; (Hoc*/Hag) × *Hoc*. Figures of natural hybrids were cited from a putative description of chromosome elimination proposed by Ogielska (2009) [20]. Interphase to Anaphase I is included in the first meiotic division and Anaphase II in the second meiotic division. Large chromosomes are composed of a set of two chromosomes. In natural hybrids, the large chromosome appears to be transmitted intact without recombination to the gametes. However, in BC-M, the large chromosome becomes fissured into two chromosomes after segregation and recombination of genomes from the female and the male.

The genomes of BC-M hybrids comprised *Hoc* and Hoc* genomes. Although the Hoc* genome inherited hemiclonally from natural hybrids comprises two to three large metacentric chromosomes, the *Hoc* and Hoc* genomes should be homologous. Genotyping with microsatellite DNA revealed that BC-M hybrids produced recombinant gametes (Table 2). In addition, the large metacentric chromosomes disappeared in the progeny of BC-M. Based on these results, the following process of fissuring in these large metacentric chromosomes is proposed (Fig 6). All of the chromosomes, including the large chromosomes, are bivalent before meiosis. When the homologous chromosomes are paired at the first meiotic division, the large metacentric chromosomes should combine with the two homologous chromosomes. The tetrad should be composed of two pairs of homologous chromosomes. After random segregation and recombination of genomes from the female and the male, the large chromosomes fissure into two different chromosomes during the second meiotic division. In this way, all of the chromosomes of the Hoc* genome is homologous to those of the *Hoc* genome, and BC-M produces recombinant gametes by undergoing meiosis.

### Derivation of microchromosomes

In addition the large metacentric chromosomes, one or two microchromosomes were also observed in natural hybrids, but not in the pure species. Microchromosomes have been observed in hybrids between colored carp (Nishikigoi), *Cyprinus carpio*, and Japanese crucian carp, *Carassius cuvieri* [41]. In that study, when colored carp was crossed with common carp *Cyprinus carpio*, it was observed that the microchromosomes disappeared during reconstruction of chromosomes during meiosis [41]. Microchromosomes were also found in the gynogenetic fish, *Poecilia formosa* [42, 43]. Since microchromosomes diversify in pigment cells, these excess chromosomes are considered to be one way of producing genetic divergence in a gynogenetic strain [44]. In *Poecilia formosa*, the microchromosomes are acquired from males due to gene leakage attributed to a failure in the normal sperm-exclusion mechanism [42, 43]. Microchromosomes found in *Hexagrammos* hybrids likely comprise the short arms of submetacentric or subtelocentric chromosomes that were torn off when the two chromosomes formed a large metacentric chromosome. In intraspecific crosses, all homologous chromosomes typically pair during meiosis; however, in hybrids, it can be difficult for some chromosomes to pair precisely due to slight differences between homoeologous chromosomes. Unlike the gynogenetic fish, *Poecilia formosa*, the microchromosomes in *Hexagrammos* hybrids may be transmitted from the hybrid genome.

### Existence of genetic factors responsible for inducing hybridogenesis

Natural hybrids comprise Hoc* and *Hag* (or *Hot*) genomes, and F_1_-hybrids comprise *Hoc* and *Hag* (or *Hot*) genomes. The former produce hemiclonal gametes, but the latter produce recombinant gametes [17]. It therefore appears that genome heterogeneity (genetic affinity) alone cannot induce hybridogenesis. BC-P hybrids have genomes that thoroughly correspond with those of natural hybrids containing the Hoc* and *Hag* (or *Hot*) genomes. BC-M hybrids produce recombinant gametes, although these gametes comprise Hoc* and *Hoc* genomes. These relationships suggest that the paternal genome is not eliminated when it meets a conspecific genome. Given the difference between *Hoc* and Hoc* genomes, genetic factors associated with hybridogenesis are probably involved in the observed Robertsonian translocation. In *Hexagrammos* hybrids, although the genetic affinity between parental species plays an important role in facilitating hybridogenesis, it is not considered to be sufficient for inducing hybridogenesis entirely. Consequently, the case described in this study does not support the balance hypothesis proposed by Moritz *et al*. (1989) [24].

In previous studies on hybridogenesis and gynogenesis, Robertsonian translocation has not been reported based on karyological observations [16, 18, 19]. At least for the *Hexagrammos* hybrid system, the genetic factors that play a significant role in normal gametogenesis are probably located near where two chromosomes undergo Robertsonian translocation. Thus, by identifying chromosomes that appear to be highly relevant to hybridogenesis, we consider that this study has advanced our understanding of (hemi-) clonal reproduction.

## Supporting information

S1 Fig Mitotic metaphase chromosome spread of F_1_-hybrid. (a) *Hoc* × *Hag*, (b); *Hoc* × *Hot*.
(TIF)

S2 Fig Mitotic metaphase chromosome spreads and karyotypes of natural hybrids Hoc*/*Hag*. (a) type 2, (b) type 3. Arrow heads show large metacentric chromosomes, and arrow shows microchromosome.
(TIF)

S3 Fig Mitotic metaphase chromosome spreads and karyotypes of natural hybrids Hoc*/*Hot*. (a) type 2, (b) type 3. Arrow heads show large metacentric chromosome and, arrow shows microchromosome.
(TIF)

S4 Fig Mitotic metaphase chromosome spreads and karyotypes of maternal backcross BC-M1 ((Hoc*/*Hag*) × *Hoc*). (a) type 2, (b) type 3. Arrow heads show large metacentric chromosome and, arrow shows microchromosome.
(TIF)

S5 Fig Mitotic metaphase chromosome spreads and karyotypes of maternal backcross. (a) BC-M2 ((Hoc*/*Hot*) × *Hoc*) type 2 Arrow heads show large metacentric chromosomes, and arrow shows microchromosome.
(TIF)
