# Supplementary figures and images for: Karyological evidence of hybridogenesis in Greenlings (Teleostei: Hexagrammidae)
Source: PLoS One. 2017 Jul 5;12(7):e0180626. doi: 10.1371/journal.pone.0180626 (PMC5498075; doi:10.1371/journal.pone.0180626)

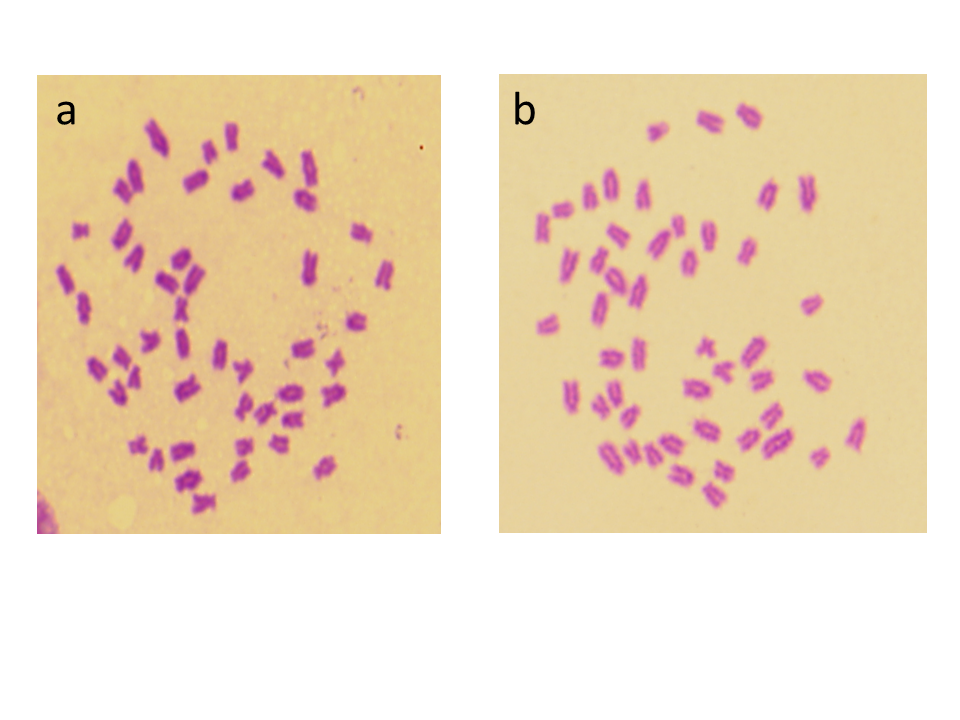

Supplement: S1 Fig — (a) Hoc × Hag, (b); Hoc × Hot. (TIF) [file pone.0180626.s001.tif]

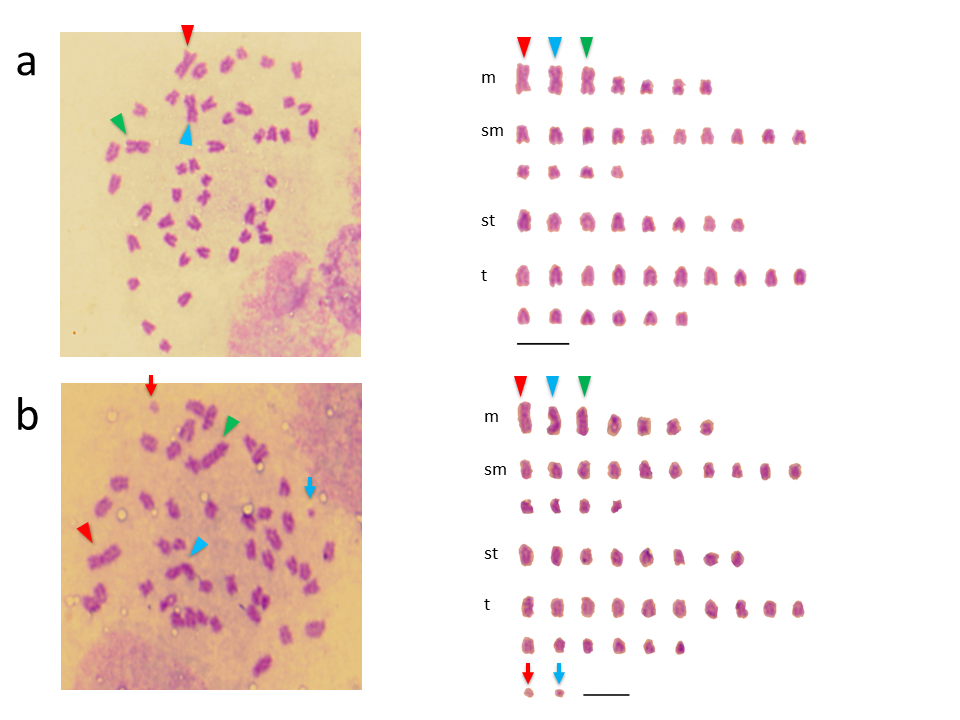

Supplement: S2 Fig — (a) type 2, (b) type 3. Arrow heads show large metacentric chromosomes, and arrow shows microchromosome. (TIF) [file pone.0180626.s002.tif]

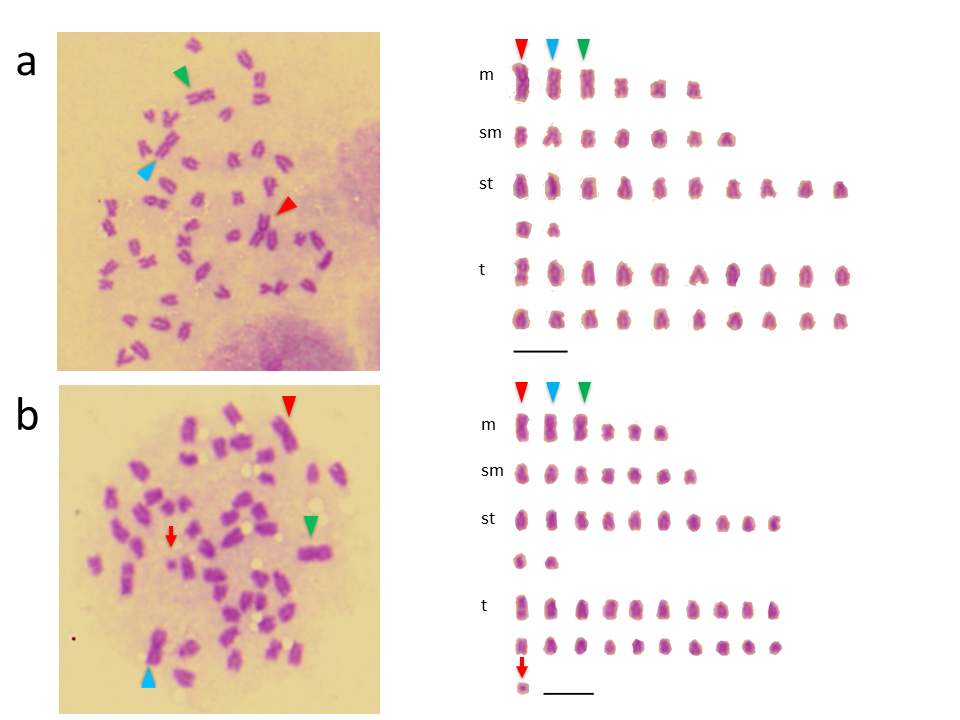

Supplement: S3 Fig — (a) type 2, (b) type 3. Arrow heads show large metacentric chromosome and, arrow shows microchromosome. (TIF) [file pone.0180626.s003.tif]

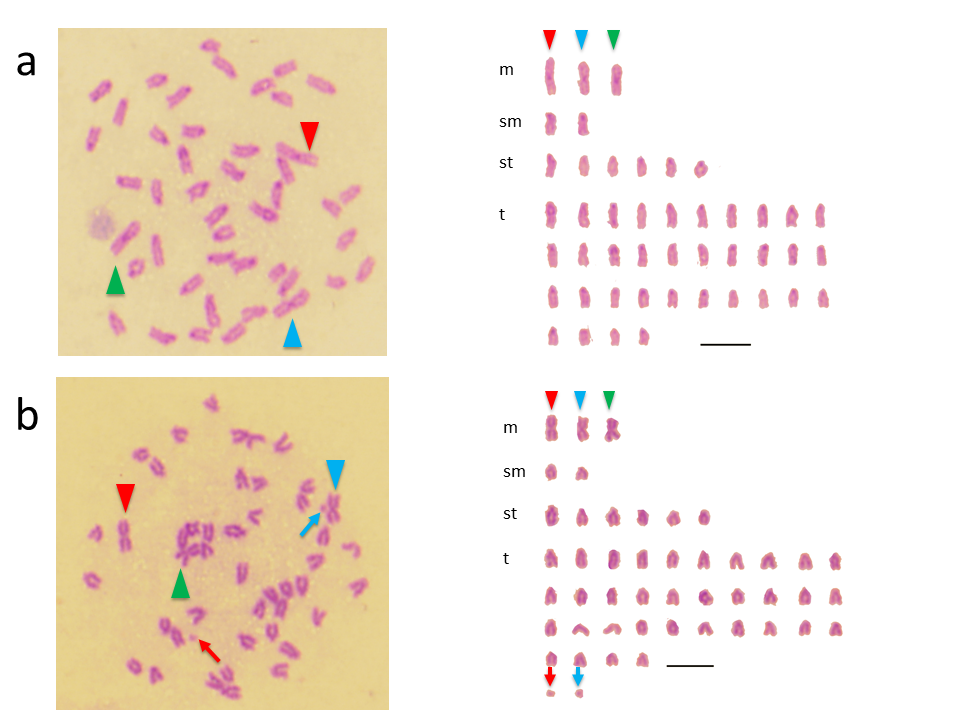

Supplement: S4 Fig — (a) type 2, (b) type 3. Arrow heads show large metacentric chromosome and, arrow shows microchromosome. (TIF) [file pone.0180626.s004.tif]

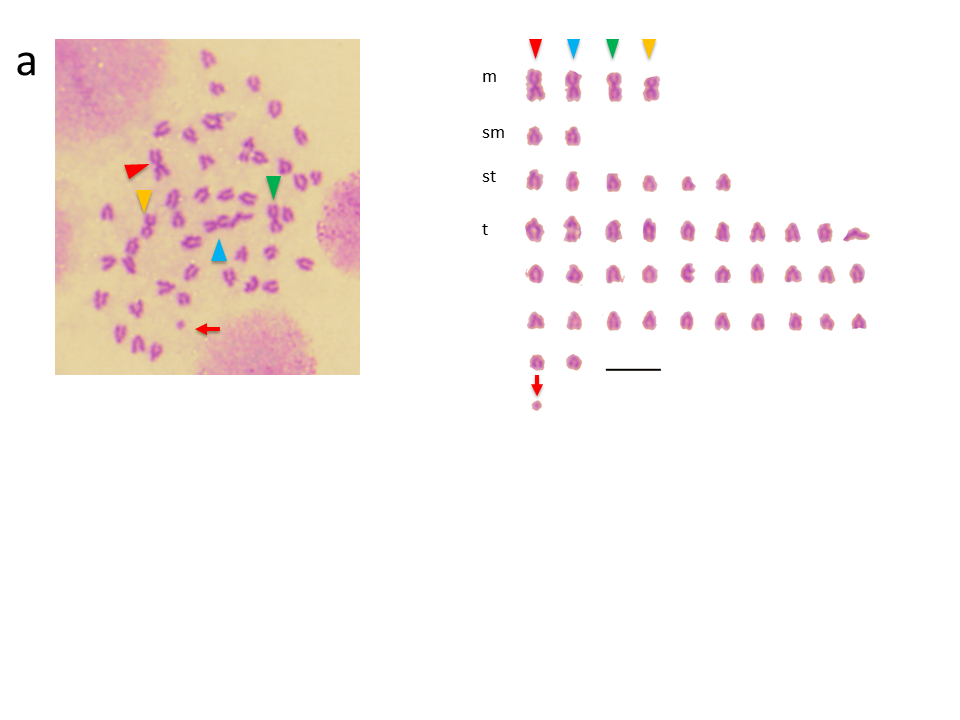

Supplement: S5 Fig — (a) BC-M2 ((Hoc*/Hot) × Hoc) type 2 Arrow heads show large metacentric chromosomes, and arrow shows microchromosome. (TIF) [file pone.0180626.s005.tif]
